# Antihypertensive Effect of Long-Term Oral Administration of Jellyfish (*Rhopilema esculentum*) Collagen Peptides on Renovascular Hypertension

**DOI:** 10.3390/md10020417

**Published:** 2012-02-15

**Authors:** Yongliang Zhuang, Liping Sun, Yufeng Zhang, Gaoxiang Liu

**Affiliations:** Research Center of Food Engineering, College of Chemistry and Engineering, Kunming University of Science and Technology, Kunming, Yunnan 650600, China; Email: kmlpsun@yahoo.com.cn (L.S.); zhangyufeng062@163.com (Y.Z.); lgx1110@126.com (G.L.)

**Keywords:** jellyfish, collagen peptide, blood pressure, angiotensin II, kidney

## Abstract

Antihypertensive effect of long-term oral administration of jellyfish (*Rhopilema esculentum*) collagen peptides (JCP) on renovascular hypertension rats (RVHs) was evaluated. The systolic blood pressure and diastolic blood pressure of the RVHs were significantly reduced with administration of JCP (*p* < 0.05), compared with model control group. However, the arterial blood pressure of normal rats showed no significant changes during long-term oral treatment with high dose JCP (*p* > 0.05). Furthermore, effect of JCP on angiotensin II (Ang II) concentration of plasma had no significance (*p* > 0.05), but JCP significantly inhibited the Ang II concentration in RVHs’ kidney (*p* < 0.05). The kidney should be the target site of JCP.

## 1. Introduction

Hypertension is one of the most common lifestyle-related diseases and has become one of the most significant problems in recent years [1]. Angiotensin-I-converting enzyme (ACE) plays an important physiological role in regulating blood pressure. It raises blood pressure by converting the inactive decapeptide angiotensin-I (Ang I) to the potent vasoconstrictor octapeptide angiotensin-II (Ang II), as well as inactivating the vasodilating nonapeptide [2]. Therefore, it is feasible to suppress blood pressure elevation by inhibiting the catalytic action of ACE. 

Synthetical ACE inhibitors, such as captopril, enalapril, and lisinopril, are effective for decreasing blood pressure. However, some undesirable side effects have been reported, including coughing, dizziness, headache, abnormal taste, and kidney and liver problems [3]. Therefore, safer alternatives are desirable. Recently, many peptides that exert ACE-inhibitory activity have been isolated from food-derived marine protein hydrolysates, such as cod [4], sea cucumber collagen [5], shrimp [6], salmon [7], and squid skin [8].

Antihypertensive peptides must be resistant to *in vivo* proteolysis once they are within the body, in order to reach the desired target tissue or organ and exert an antihypertensive effect [9]. Therefore, it is necessary to confirm the antihypertensive effect of orally administrated peptides on renovascular hypertension rats (RVHs) or spontaneously hypertensive rats (SHR). The antihypertensive effects *in vivo* of peptides prepared from various sources have been reported in previous works [5,10,11], but most of them did short-term experiments with rats within 24 h. In the present study, a long-term antihypertensive experiment was performed.

Previously, we isolated and purified peptides from jellyfish collagen (JCP). The IC_50_ of ACE-inhibitory activity of JCP was 43 μg/mL *in vitro* [1]. In this study, the antihypertensive of JCP was determined by RVHs *in vivo*. The effect of JCP on the blood pressures and heart rates was investigated. Furthermore, the concentrations of Ang II in plasma and kidney were quantitated.

## 2. Results and Discussion

### 2.1. Blood Pressure and Heart Rate

**Figure 1:**
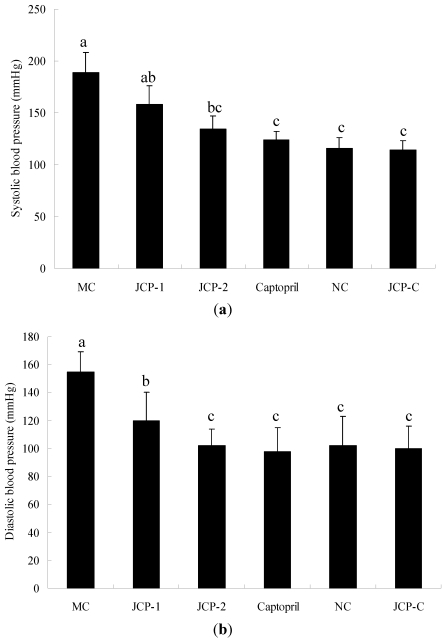
Effect of jellyfish (*Rhopilema esculentum*) collagen peptides (JCP) on systolic blood pressure (SBP) (**a**) and diastolic blood pressure (DBP) (**b**) of renovascular hypertension rats (RVHs) after oral administration for 30 days. Values are mean ± SD (*n* = 6). Different letter indicated significant differences (*p* < 0.05). MC group: laparotomy and isolation of the left renal artery with clip placement (2K1C) with oral administration of 0.9% saline solution; JCP-1 group: 2K1C with oral administration of JCP (25 mg/kg bw); JCP-2 group: 2K1C with oral administration of JCP (100 mg/kg bw); Captopril group: 2K1C with oral administration of captopril (5 mg/kg bw); NC group: sham surgery (laparotomy and isolation of the left renal artery without clip placement) with oral administration of 0.9% saline solution; JCP-C group: sham surgery with oral administration of JCP (100 mg/kg bw).

Antihypertensive effect of JCP was evaluated by measuring changes in systolic blood pressure (SBP) and diastolic blood pressure (DBP) of RVHs. Figure 1 showed the SBP and DBP obtained after 1 month of oral administration of the different products, respectively. Compared with MC group, the average SBP reductions in all groups were, in descending order: Captopril group (65.2 mmHg), JCP-2 group (55.5 mmHg) and JCP-1 group (32.0 mmHg). The antihypertensive effect improved with a JCP dosage increase. The average DBP of JCP-1 group (120.2 mmHg) and JCP-2 group (102.4 mmHg) decreased by 34.8 and 52.6 mmHg compared with MC group, respectively. DBP showed no significant difference between JCP-2 group and captopril group (*p* > 0.05). Interestingly, the SBP and DBP of JCP-C group showed no significant changes during long-term oral treatment with high dose JCP (*p* > 0.05). These results indicated that JCP had good long-term antihypertensive effects on RVHs, but had no effects for the blood pressures of normal rats. 

**Figure 2:**
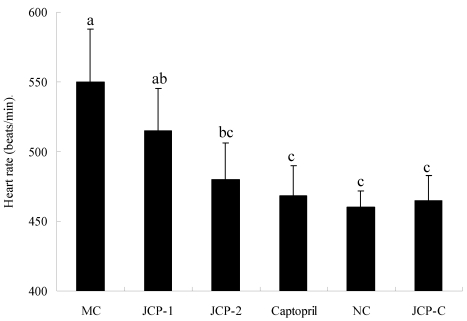
Effect of JCP on heart rate of RVHs after oral administration for 30 days. Values are mean ± SD (*n* = 6). Different letter indicated significant differences (*p* < 0.05). MC group: laparotomy and isolation of the left renal artery with clip placement (2K1C) with oral administration of 0.9% saline solution; JCP-1 group: 2K1C with oral administration of JCP (25 mg/kg bw); JCP-2 group: 2K1C with oral administration of JCP (100 mg/kg bw); Captopril group: 2K1C with oral administration of captopril (5 mg/kg bw); NC group: sham surgery (laparotomy and isolation of the left renal artery without clip placement) with oral administration of 0.9% saline solution; JCP-C group: sham surgery with oral administration of JCP (100 mg/kg bw).

As shown in Figure 2, the heart rates of the MC group were significant higher than the captopril group and NC group (*p* < 0.05). The heart rate of JCP-C group was not different from NC group (*p* > 0.05), and the heart rats of JCP-2 group had no significance with captopril group and NC group (*p* > 0.05). 

Human tissues are exposed to bioactive peptides via the systemic circulation. However, before bioactive peptides reach tissues, peptides are substantially hydrolyzed during small intestinal passage and absorption [12]. The digestive system *in vivo* is capable of handling a wide range of protein sources and the cascade of gastrointestinal proteolytic and peptidolytic enzymes very efficiently degrades proteins from their quaternary structure into single amino acids. Some peptides failed to show antihypertensive activity after oral administration to spontaneously hypertensive rats, in spite of their *in vitro* activity [13]. Because peptides are prone to extensive hydrolysis in the gastrointestinal tract by stomach, small intestinal, and brush border peptidases, so bioactive peptides must be absorbed from the intestine intact and be resistant to degradation by plasma peptidases to reach the target sites [14], so antihypertensive peptides have limited application if they have no oral activity [15]. Phe-Lys-Gly-Arg-Tyr-Tyr-Pro isolated from the thermolysin digest of chicken muscle showed an *in vitro* IC_50_ value of 0.55 mM, but no antihypertensive activity could be observed after oral administration to SHR [16]. Vercruysse *et al.* studied enzymatic hydrolysates of insect protein had high ACE inhibitory activity [17]. Ala-Val-Phe and Val-Phe were the key peptides in the hydrolysates. The ACE inhibitory activity of Val-Phe was higher than that of Ala-Val-Phe, and in organ bath experiments using rat aorta, Val-Phe showed ACE inhibitory, while Ala-Val-Phe did not. However, single oral administration to spontaneously hypertensive rats led to a significant decrease in blood pressure for both peptides, because Val-Phe was released by *in vivo* peptidases from Ala-Val-Phe [18]. Our previous study showed JCP had potent ACE inhibitory activity *in vitro* with an IC_50_ value of 43 μg/mL [1]. In this study, antihypertensive activity of JCP was evaluated *in vivo*. After the oral administration of JCP, the SBP and DBP of RVHs significantly decreased (*p* < 0.05), indicating that JCP had an intense effect on the reduction of blood pressure *in vivo*. Therefore, some peptides of JCP may have resistance to gastrointestinal enzymes and could be absorbed in their intact active form to lower the blood pressure.

### 2.2. Angiotensin II Concentration

**Figure 3:**
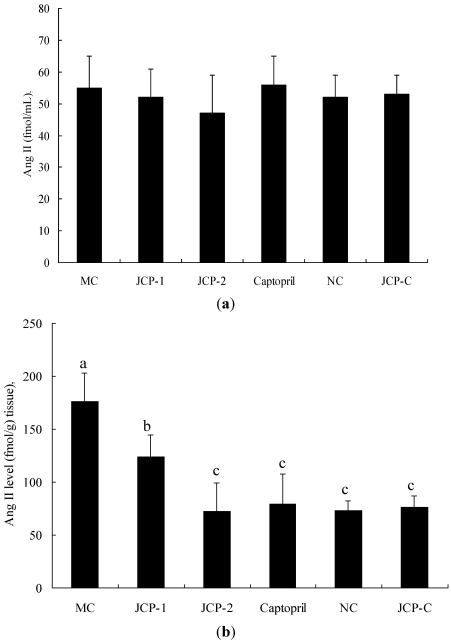
Effect of JCP on the Ang II concentrations of plasma (**a**) and kidney (**b**) of RVHs after oral administration for 30 days. Values are mean ± SD (*n* = 6). Different letter indicated significant differences (*p* < 0.05). MC group: laparotomy and isolation of the left renal artery with clip placement (2K1C) with oral administration of 0.9% saline solution; JCP-1 group: 2K1C with oral administration of JCP (25 mg/kg bw); JCP-2 group: 2K1C with oral administration of JCP (100 mg/kg bw); Captopril group: 2K1C with oral administration of captopril (5 mg/kg bw); NC group: sham surgery (laparotomy and isolation of the left renal artery without clip placement) with oral administration of 0.9% saline solution; JCP-C group: sham surgery with oral administration of JCP (100 mg/kg bw).

The Ang II concentrations of the plasma and kidney were investigated. Figure 3 depicted changes in the plasma and kidney Ang II levels after the JCP dosing. The concentration of Ang II in plasma had no significance during the period (*p* > 0.05). This suggested that JCP was not directly associated with an inhibition of plasma ACE. Compared with NC group, JCP-1 group and JCP-2 group significantly decreased Ang II concentration in kidney during long-term oral treatment (*p* < 0.05), and JCP-2 group had no significance with captopril group and NC group (*p* > 0.05). Interestingly, during long-term oral treatment with high dose JCP (100 mg/kg), the Ang II concentrations of the plasma and kidney of JCP-C group were not different from NC group (*p* > 0.05).

Classically, the rennin-angiotensin system plays an important role in regulating arterial pressure [19]. ACE is a nonspecific dipeptidyl carboxypeptidase associated with it. This enzyme increases blood pressure by converting the Ang I into the Ang II [20]. Ang II has many important actions, including increasing arterial pressure, increasing sodium and fluid retention, enhancing sympathetic adrenergic function and causing cardiac and vascular remodeling [21]. Inhibition of ACE is believed to lower blood pressure, since it reduces the production of Ang II. Experimental Renovascular Hypertension is characterized by significant up-regulation of intrarenal Ang II levels, which is believed to play an important pathogenetic role in the development and maintenance of hypertension in RVH. Researchers thought that the prolonged antihypertensive action of ACE inhibitors might be related to persistent inhibition of ACE activity and Ang II concentrations of tissues, such as vascular wall and kidneys [22,23]. The antihypertensive activities of captopril are known to be related to the inhibition of ACE activities and Ang II concentration in such organs as the lung, kidney, aorta, and brain [24]. The present study investigated the role of Ang II production in the plasma and kidney. Our results showed the concentration of Ang II in plasma had no significance during long-term oral administration JCP (*p* > 0.05), but the concentration of Ang II in kidney significantly decreased in JCP groups (*p* < 0.05). So, the kidney should be the target site of JCP. Miguel *et al.* [25] indicated that ACE inhibitors with different molecular structures had different metabolism pathways and tissue distribution. These characteristics determined an ACE inhibitory effect in tissue and thus exhibited different antihypertensive effects. Lin *et al.* [8] reported the administration of Captopril produced a significant decrease in plasma concentrations of Ang II (*p* < 0.05), but squid skin gelatin hydrolysates had no statistically significance (*p* > 0.05). Matsui *et al.* [26] had demonstrated that Val-Tyr greatly affected tissue Ang II in the lung, heart, kidney, aorta and mesenteric artery, whereas no suppression was observed in the plasma. 

## 3. Experimental Section

### 3.1. Materials

Jellyfish collagen peptides (JCP) were prepared by the previous study. Captopril was purchased from Sigma Chemical Co. (St. Louis, MO). All other reagents used in this study were analytical grade.

### 3.2. Animal Treatment

The Wistar strain male rats (SPF, 150 ± 20 g) were fed ad libitum and housed under conventional conditions at a controlled temperature (23 ± 2 °C), humidity (55 ± 10%), and light (12 h light/12 h darkness). All animal treatments were strictly in accordance with international ethical guidelines and the National Institutes of Health Guide on the Care and Use of Laboratory Animals. A two-kidney, one-clip (2K1C) rat model was used to induce RVH. A midline laparotomy was used for placement of a partially occlusive, a silver clip (0.2 mm internal diameter) on the left renal artery of 2K1C rats [27]. 

The rats were grouped as model control (MC), negative control (NC), positive control (captopril), JCP-1 (25 mg/kg bw), JCP-2 (100 mg/kg bw) and JCP-C (100 mg/kg bw) (*n* = 6 in each group). Sham surgery (laparotomy and isolation of the left renal artery without clip placement) was used as a negative control and JCP-C group underwent the sham surgery with oral administration of JCP (100 mg/kg bw). NC group and MC group were administrated with 0.9% saline solution (5 mL/kg bw). Captopril (5 mg/kg bw), a known ACE inhibitor, served as a positive control group. JCP-1 group (25 mg/kg bw), JCP-2 group (100 mg/kg bw) and JCP-C group (100 mg/kg bw) were daily given the JCP dissolved in the same volume of saline solution *via* gastric intubation for 30 days.

### 3.3. Measurement of Blood Pressure and Heart Rate

Thirty days after oral sample administrations, the rats were anesthetised with sodium pentobarbital (40 mg/kg bw). A catheter was inserted into the left carotid artery to measure mean arterial pressure and heart rate with a pressure transducer coupled to computer recorder (GY-6088, Kaifeng-Huanan, China).

### 3.4. Determination of Angiotensin-II (Ang II) Concentration

The plasma and kidney were collected to measure Ang II concentration. The concentrations of Ang II were determined by Ang II radioimmunoassay kits (Beijing North Institute of Biological Technology, China) [8].

### 3.5. Statistical Analysis

Results were expressed as the mean ± SD. Data were analyzed by one-way analysis of variance (ANOVA) using SPSS (version 11.0, Chicago, IL, USA) and the differences between the means assessed using Duncan’s multiple range test. A *p* value of <0.05 was taken as the level of statistical significance.

## 4. Conclusions

Antihypertensive effect of long-term oral administration of jellyfish collagen peptides (JCP) on renovascular hypertension rats (RVHs) was evaluated *in vivo* in this study. The results showed JCP was a safe selection to decrease blood pressure. After oral administration of JCP, the systolic blood pressure and diastolic blood pressure of RVHs significantly decreased (*p* < 0.05), indicating that JCP had an intense effect on the reduction of blood pressure *in vivo*. Furthermore, JCP showed antihypertensive effects through inhibiting the Ang II concentrations in the kidney, and the kidney should be the target site of JCP. 
